# Emergency nurses' experiences of caring for brought‐in‐dead persons and their relatives—a qualitative study

**DOI:** 10.1111/jan.16371

**Published:** 2024-08-06

**Authors:** D. G. Bove, S. S. Simonsen, S. F. Herling, H. Timm

**Affiliations:** ^1^ Centre for Nursing University College Absalon Roskilde Denmark; ^2^ Department of People and Technology Roskilde University Roskilde Denmark; ^3^ The Neuroscience Centre Copenhagen University Hospital, Rigshospitalet Copenhhagen Denmark; ^4^ Department of Clinical Medicine Copenhagen University Copenhagen Denmark; ^5^ The University Hospitals Centre for Health Research Rigshospitalet Copenhagen Denmark; ^6^ National Institute of Public Health University of Southern Odense Denmark; ^7^ Faculty of Health Sciences University of the Faroe Islands Torshavn Faroe Islands

**Keywords:** already dead, dead on arrival, emergency care, emergency nursing, moral stress, palliative care, sudden death

## Abstract

**Aim:**

To explore how emergency nurses experienced caring for brought‐in‐dead persons and their relatives, and what hindered or facilitated this care in an emergency setting.

**Design:**

A qualitative study using Interpretive Description.

**Methods:**

Data were collected as individual interviews with 13 nurses at seven Danish emergency departments from February to June 2023.

**Findings:**

Our analysis revealed the overarching theme ‘Navigating the complexities of providing holistic care in a constrained environment’, covering five sub‐themes: (1) An important yet not recognized nursing task; (2) Pending care needs of the living and the dead; (3) No physical or mental room for the brought‐in‐dead persons; (4) Utilizing personal experiences in the absence of formal education and training and (5) Navigating professionalism and empathy.

**Conclusion:**

Emergency departments posed unique challenges in providing care to brought‐in‐dead persons and their relatives.

**Implications for the Profession:**

The unrecognized nature of caring for brought‐in‐dead persons and their relatives suggests a universal undervaluation of this care in emergency departments.

**Impact:**

Care for brought‐in‐dead persons and their relatives is neither recognized nor evidence‐based. This study initiates a discussion of the circumstances for delivering care for persons brought‐in‐dead and has an impact on nurses and nursing leaders employed in emergency departments.

**Reporting Method:**

The Consolidated Criteria for Reporting Qualitative Research (COREQ).

**Patient or Public Contribution:**

None.

**What does this paper contribute to the wider global clinical community?**

There is a need to discuss whether care for brought‐in‐dead persons and their relatives should be the responsibility of emergency nurses or not. If so, it is crucial to ensure that the task is recognized and that the nurses have the education, support and conditions enabling them to provide this care in a morally and professionally justifiable manner.

## 1 INTRODUCTION

Emergency Departments (EDs) are primarily designed to save lives, with ED nurses being specifically educated and trained in acute care and life‐saving skills (Giles et al., 2019). However, the ED nurses also have the responsibility of caring for persons who are brought‐in‐dead (BID) and their relatives. BID persons are persons who are dead on arrival and have died unexpectedly at home, in the street, at work, in traffic or on the way to the hospital. BID persons are globally received and cared for in EDs (Bove et al., 2020). Presently, knowledge about how to provide care for BID persons and their relatives in an ED setting is notably limited (Aquino et al., 2022; Giles et al., 2019). Therefore, this study aims to explore how ED nurses experience caring for BID persons and their relatives and what may hinder or facilitate this care in an ED.

## 2 BACKGROUND

EDs are designed and organized to save lives, with ED nurses being educated and trained in acute care and life‐saving skills (Hunter et al., 2018a, 2018b). However, in addition to caring for acutely ill patients, caring for BID persons and their relatives is also a responsibility for ED nurses. BID is a term used in medical and paramedical contexts when a person is found dead unexpectedly outside a hospital or dies after unsuccessful resuscitation in the ambulance or in a trauma room before being admitted. The terms ‘death on arrival’, ‘sudden dead’ or ‘already dead’ are used synonymously in the literature (Alarhayem et al., 2017; Bove et al., 2021; Sefton et al., 2023).

Due to the often unexpected nature of BID persons' deaths, relatives frequently wish to visit the ED to see and be with the deceased one last time (Bove et al., 2022; Merlevede et al., 2004). In a previous study, we explored the BID relatives' experiences of seeing and saying goodbye to a BID person in a Danish ED setting. We found that the relatives' experiences were influenced by both internal and external chaos and that the external chaos in the ED amplified their feelings of internal turmoil. Despite the short duration of the BID relatives' stay in the ED, the visit and care from the ED nurses were described as being significant to them and their grieving process (Bove et al., 2022).

In the past decade, there has been a growing recognition of the importance of providing palliative care in EDs and the subsequent need for educating and training ED staff in the necessary competences and skills (Chang et al., 2022; Gage et al., 2023). The World Health Organization (WHO) and palliative care stakeholders recommend that all nurses and nursing‐students are educated in palliative care, as it is fundamental to nursing practice (De Campos et al., 2022; Murnane et al., 2023; Taheri‐Ezbarami et al., 2024; World Health Organisation (WHO), 2024). Palliative care education and the development of core competences and skills for undergraduate nursing students are priorities to ensure that graduate nurses are equipped with the knowledge and skills to deliver safe and competent palliative care in all kinds of settings (Cavaye & Watts, 2014; Durojaiye et al., 2023; Murnane et al., 2023; Powers, 2017; Taheri‐Ezbarami et al., 2024).

In Denmark, palliative care is part of the nursing students' curriculum, but it is not included in the additional education at the diploma level in emergency nursing. Consequently, Danish ED nurses seldom possess additional skills or education in palliative care.

Although the focus in EDs is on curative and life‐saving interventions, death is also a daily occurrence, and most nurses have some experience caring for palliative patients and their bereaved relatives (Aquino et al., 2022b; Giles et al., 2019; Martí‐García et al., 2023). Additionally, nurses often have experience caring for bereaved relatives after unsuccessful resuscitation attempts (Bradley, 2021).

While ED nurses can draw on their pre‐graduate education in palliative care and their palliative care experience from the ED, palliative care in the ED still presents unique challenges due to the primary emphasis on patient flow and life‐saving interventions (Aquino et al., 2022a; Díaz‐Cortés et al., 2018; Fernández‐Sola et al., 2017; Giles et al., 2019). Furthermore, we have observed that nurses who choose to work in EDs often do so because they are attracted to the dynamic and fast‐paced nature of acute care, which requires a different set of skills and may not prominently feature palliative care competencies (Aquino et al., 2022a; Eriksson et al., 2018; Sausman et al., 2023a).

Research on palliative care within ED contexts predominantly centres on strategies to prevent patient admissions to EDs or reduce their length of stay in the ED (Nordt et al., 2023; Sausman et al., 2023a). Although there is an objective shift from acute and life‐saving care to palliative care, healthcare professionals in EDs are still obliged to deliver a form of problem‐solving treatment, enabling patients to either return home, be transferred to another kind of institution, or be admitted to a hospital ward (Bayuo et al., 2022; Giles et al., 2019; Prachanukool et al., 2023).

It is well known from specialized palliative care research that a relationship with the healthcare professionals and surroundings with privacy, dignity, time and space to be with the dying or deceased person is of importance to the relatives and their subsequent grieving process (Díaz‐Cortés et al., 2018; Hajradinovic et al., 2018; Prachanukool et al., 2023). However, in an ED setting, ED nurses, the BID persons and the relatives do not have any pre‐existing relationships (Aquino et al., 2022a). In addition, the environment in EDs often offers little room for privacy, time and space, affecting the relatives' opportunity to be with the BID person and the nurses' opportunity to care for them during their stay in the ED (Beckstrand et al., 2012, 2019). Nurses perceive the death of a patient as a natural part of their work in the ED. However, dealing with relatives after a death and witnessing the intensity of their grief is described by both novice and experienced nurses as one of the most challenging aspects of their profession (Hogan et al., 2016; Mayer, 2017).

BID‐relatives, due to the unexpected and potential traumatic death of a loved one, find themselves in a particularly vulnerable situation, with a high risk of developing complicated grief reactions (Heeke et al., 2017; Şimşek Arslan & Buldukoğlu, 2023; Szuhany et al., 2021; Wilson et al., 2022). It is also known that relatives of persons who committed suicide experience stigma, guilt and shame, which negatively affect their grieving process (Pitman et al.,  2018; Zavrou et al., 2023).

Qualitative studies have stated that it was helpful for bereaved relatives to see the body after a sudden death like a suicide or a traumatic death and that the bereaved persons seldom regretted doing so (Howarth, 2010; Merlevede et al., 2004). The reason for relatives to see the body of a deceased person was to identify the deceased, care for the deceased and to say goodbye (Howarth, 2010; Tarassenko, 2010). It was of great importance to the relatives that they were carefully informed in advance about the appearance of the deceased, any visible damage to the body and how this viewing could be altered, for example, by shielding or hiding damaged parts of the body (Howarth, 2010; Omerov et al., 2014; Tarassenko, 2010). No studies have been identified describing the viewing of the body after the sudden, but non‐traumatic, death of an older person, although we in a former study have illustrated that the mean age of a brought‐in‐dead person in a Danish ED is 71 years and that the majority of brought‐in‐dead persons were found dead or dying in their home (80%) and often by a spouse, cohabitant or son/daughter (54%) (Bove et al., 2021).

To alleviate suffering and prevent prolonged grief, it is of utmost importance that the relatives of BID persons receive care and support as early as possible (Carlsson et al., 2022; Mughal et al., 2024). Often, the first and perhaps the only healthcare professionals BID relatives encounter are the ED nurses. While we have previously described what relatives seek in support in such situations (Bove et al., 2022), we still lack evidence‐based knowledge about how ED nurses experience caring for BID persons and their relatives and what may hinder or facilitate this care in an acute setting.

## 3 THE STUDY

### 3.1 Aim

This study aimed to explore how ED nurses experienced caring for BID persons and their relatives, and what hindered or facilitated this care in an ED setting.

### 3.2 Design

The design was qualitative and used interpretive description (ID) methodology (Thompson Burdine et al., 2021; Thorne, 2016). This study was part of a larger project with the overall aim of improving care for BID persons and their relatives. The project and its methodological reflections were described in detail in a qualitative research protocol (Bove et al., 2020).

Data was constructed through semi‐structured interviews with 13 ED nurses from seven different Danish EDs. The analysis was thematic as described by Braun & Clarke and the recommendation of the ID methodology (Braun & Clarke, 2006, 2019; Byrne, 2022; Clarke & Braun, 2017; Thompson Burdine et al., 2021; Thorne, 2016). The study complied with the Consolidated Criteria for Reporting Qualitative Research (COREQ) (Tong et al., 2007).

### 3.3 Participants

The study participants were recruited from seven EDs located in the Capital Region of Denmark and Region Zealand. In Denmark, we have 21 hospitals with emergency departments. While they are all organized differently, they all together treat more than 1.2 million patients annually.

The sampling strategy was convenient, with maximal variation in terms of gender, age and experience as an ED nurse. The participating ED nurses were appointed and recruited through their leaders (chief nurses), who were contacted and informed about the research project by mail or telephone by the first author. All contacted leaders replied; however, approximately half of the ED nurses declined to participate due to a lack of free time to engage in the research project.

### 3.4 Data collection

All interviews (except one) were conducted in a meeting room in the actual ED during the period from February to June 2023. One interview was conducted by phone. The interviews lasted from 25 to 90 min (mean 45 min). The interviews were conducted by the second author, who is an experienced interviewer.

The topics of the interview topic guide were found through discussions between the first and second authors and anchored in the existing literature and the first authors' clinical experience in providing care for BID persons and their relatives. The interview technique was open‐ended with follow‐up questions related to the participants' responses (Table 1).

**TABLE 1:** Topics of the interview guide with keywords.

Topics	Keywords for inspiration
Situations and episodes	Care experiences, memorable BID situations, descriptions in time and process
Physical framework and practices	Room and premises, atmosphere and artefacts, catering for the relatives, clothes, colours, bibles/hymn books, open windows or other rituals
Debriefing	Reactions of nurses, coping strategies, debriefing, self‐care (nurses), support and leader support
Culture and care practice	The way you talk about BID, the relatives, grief and death. Planning, prioritization, dilemmas, reactions of the relatives, support and care
Education and training	What are you taught and how? Training at the ED or at school. Available material, guidelines, instructions, and so forth
Organization, planning and collaboration	Culture, work environment, responsibility, planning of tasks/BID care, collaboration with physicians, nursing assistants and other health professionals

### 3.5 Ethical considerations

All participants were informed, both in writing and verbally, about the aim of the study and its voluntary nature. All participants signed an informed consent form before the interview. The participants' anonymity and the confidentiality of the data were ensured, and the study followed the guidelines of the Danish Data Protection Agency and the European Union's General Data Protection Regulation. The study was conducted in accordance with the Declaration of Helsinki. According to Danish law and the Ethics Committee (https://en.nationaltcenterforetik.dk/) in Denmark, no formal approval applies to qualitative studies in general. Further ethical reflections are described in the research protocol (Bove et al., 2020).

### 3.6 Data analysis

In line with Braun and Clarke's six‐step approach (Braun & Clarke, 2006, 2019; Byrne, 2022), we started by getting familiar with the data through reading and re‐reading the transcripts and by organizing the text in broad codes. This first step of the analysis was primarily led by the first and second authors. In the subsequent analysis steps, all authors participated equally actively. During this process, each researcher made descriptive notes and codes. These notes were compared and discussed in the research group preceding the first concept map and, afterward, the first tentative themes. The themes were further developed and refined through researcher triangulation and questions like ‘So what’ and ‘What does this mean’. The research group met several times during this process to discuss and reach an agreement about the final themes. We did not use any software to organize or code the material. Instead, we used paper, post‐it notes and a large whiteboard. Our protocol initially outlined the use of NVivo for data organization and coding (Bove et al., 2020). However, as not all researchers had access to NVivo at the start of the analysis, we began the process without it. Our analysis process and working method proved to be so efficient that at no point did we find NVivo could contribute to or strengthen the analysis, which is why we deviated from our original plan to use NVivo.

### 3.7 Rigour

Trustworthiness, encompassing credibility, transferability, dependability and confirmability, is a crucial criterion for determining the rigour of a qualitative study (Tobin & Begley, 2004). The ID methodology provided a theoretical framework that helped us scaffold and structure the study (Thorne, 2016). To elaborate on our methodological considerations in detail, thereby increasing the study's trustworthiness, these reflections were further described in a qualitative study protocol (Bove et al., 2020).

The entire research process, from study design to analysis and conclusion, was carried out through close collaboration and extensive discussions within our interdisciplinary research team. This collaborative approach ensured that diverse perspectives were considered, enriching the analysis and interpretation of the data. Transferability was addressed by providing a rich description of the research context, participants and findings, allowing readers to determine the applicability of the results to other settings. Dependability was ensured through a systematic and transparent research process. An audit trail was established, documenting every step of the study, from data collection to analysis and conclusions.

Throughout all stages of the research, we have persistently worked to integrate and articulate credibility, thereby enhancing ‘representative credibility’, ‘analytic logic’ and ‘interpretive authority’ in our findings (Thorne, 2016). By systematically addressing these elements of trustworthiness, we have endeavoured to produce a rigorous and credible qualitative study that contributes meaningfully to the existing body of knowledge.

## 4 FINDINGS

We conducted 13 interviews with ED nurses. The majority of the participants were female (85%). The mean age was 46 years (range 31–60), and the participants had an average experience working in an ED of 14 years (range 5–23). The participants had not received any additional training or education in palliative care or bereavement support. Out of the 13 participants, seven came from Region Zealand and six from the Capital Region of Denmark. Age, gender and experience of ED nurses are shown in Table 2.

**TABLE 2:** Characteristics of the participating nurses (*n* = 13).

ID	Name*	Age (year)	Gender	Experience as ED nurse (year)
1	Camilla	36	F	6
2	Maria	56	F	5
3	Line	58	F	20
4	Anne	56	F	20
5	Sarah	52	F	18
6	Mona	60	F	20
7	Julie	41	F	9
8	Louise	42	F	10
9	Mia	37	F	9
10	Kim	54	M	23
11	Pernille	31	F	8
12	Morten	32	M	6
13	Mette	47	F	23

Abbreviations: F, female; M, male.

* All names are fabricated.

Our analysis revealed an overarching theme ‘Navigating the Complexities of Providing Holistic Care in a Constrained Environment’, which is detailed through five sub‐themes (Table 3). This theme captures how ED nurses, despite challenging circumstances, adeptly navigate the complexities and dichotomies between the fast‐paced, outcome‐oriented nature of emergency care and the detailed, empathetic approach required for BID care.

**TABLE 3:** List of theme and sub‐themes.

Navigating the complexities of providing care in a constrained environment
An important yet not recognized nursing task
Pending care needs of the living and the dead
No physical or mental room for the BID persons
Utilizing personal experiences in the absence of formal education and training
Navigating professionalism and empathy

### 4.1 An important yet not recognized nursing task

The ED was open all day: 24–7, and the nurses described how they received and cared for a large, thus unpredictable, and diverse group of patients. The BID persons constituted a small fraction of the total number of ED patients, although BID persons were present daily in all the included EDs. Although the nurses described how they provided care for the BID persons and their relatives, this care was not registered or documented anywhere, hence seeming invisible and not valued.

…the deceased don't count as a patient requiring care because they are dead. But for us, it is some of the patients who require the most care, coverage, and precision. We have only one shot at securing a worthy situation for the diseased and for the relatives (Line, 58 years).

Providing care for the BID person and relatives was described as a significant responsibility where the nurses felt that they could make a meaningful difference in the grieving process for the relatives. It was also a task that was given priority by the nurses, with one nurse stating it as *the most important task in the ED* (*Anne*, *56 years*).

### 4.2 Pending care needs of the living and the dead

However, the harsh realities of the ED sometimes necessitated a re‐prioritization of tasks. The nurses described how working conditions and the workload in an ED were characterized by being unpredictable and changeable within min/h depending on the number and severity of the acute admitted patients. In addition to the acute admitted patients, the arrival of BID persons was also unpredictable and always unplanned. Receiving one or more BID persons at a busy time in the ED was perceived as highly chaotic and disturbing, taking time and focus from other more acute nursing tasks. The nurses described how the primary objective in an ED is ‘to save lives’, and that it is an unspoken rule ‘that the needs of the living always take precedence over the deceased’.

The prioritizing of life‐saving assignments is shared with other health professionals, such as physicians. In contrast, the care for BID persons and their relatives was a responsibility that fell solely on the nurses. The nurses described this as a dilemma. On one hand, they wished to be available and care for the BID relatives. On the other hand, the urgent needs of acutely ill patients required their immediate attention and nursing skills.

If we have to choose between saving lives in the trauma rooms and being with the relatives to brought in dead persons, we choose saving lives first (Maria, 56 years).

Even though the nurses did not doubt or question the prioritization of ‘living over the dead’, it was still described as difficult, being in situations where they actually had to opt out or delay the care for the BID persons and their relatives. The nurses expressed an awareness of having only one attempt to make the visit and time spent with the BID person ‘as good as possible’ for the relatives. However, when the ED was full of acute patients and the healthcare professionals were occupied with ‘saving lives’, the nurses found it difficult to find the time to care for the BID persons and their relatives. The nurses described how it was often a time‐consuming task to be with the relatives during their stay in the ED, and how they did not want to rush the relatives into saying goodbye in the ED. The perceived care dilemma was underscored by the emotional impact it had on the nurses. Regardless of their level of experience, several nurses were emotionally moved and shed tears during the discussion about this subject.

### 4.3 No physical or mental room for the BID persons

The nurses described the unpredictability concerning the cause of death, family relationships and acute grief reactions as complicated, and when added to the chaotic and busy ED context, that only contributes to increasing the complexity of care further. Typically, nurses only received a short notice that a BID person would arrive within 30 min or less. The nurses described how it was a condition, that it was unknown and unpredictable how many BID persons or relatives would arrive in the ED during a shift. In addition, the state and condition of the relatives and their derived need for support and care remained unknown and unpredictable until their actual arrival.

If we sense that the relatives need to stay with the deceased for a longer time or maybe return to the ED to see the deceased again, we provide that care… so, we try to create an environment and peace for the relatives to say goodbye in a good way. It could be in the case of a traffic‐related death of a young man for example (Line 58, years).

The ED context and derived work conditions and physical frameworks were described as barriers for nurses providing BID care. For example, the room in the ED where the BID relatives could be with the deceased was described as a room that did not offer any kind of comfort or dignity and, in addition, often located a little ‘out of the way’. Some nurses expressed how they were embarrassed by the appearance and location of the room and how they regretted not having the opportunity to have flowers in the room or to be able to light a candle.

… and the presentation rooms are…, very sad and very… without emotion [sterile ]… unlike when you are in a care center or rehabilitation center (Maria, 56 years).

The nurses described how an ED with constant busyness and high patient flow was in no way a suitable environment for BID persons and their relatives.

… we can contain a large group of relatives… but … we had a really busy day with a lot, a lot of incoming patients. And a lot of strain on our trauma rooms. But then there is a younger man, as I call it, who has a fall at his work, goes into cardiac arrest, and dies. Attempts were made to resuscitate at the workplace. No luck, and he gets in here already dead… Of course, he has a huge family who are deeply shocked and are crying. And they walk among the patients in the corridors and us who are in a hurry. (Maria, 56 years)

Some nurses experienced that the BID persons and their relatives disrupted the acute patients and busy staff in the ED, and some nurses stated how it did not make any sense to them that BID persons and their relatives were the responsibility of EDs.

### 4.4 Utilizing personal experiences in the absence of formal education and training

Although all nurses frequently encountered BID persons and their relatives, they had no education or training in caring for them. Due to a lack of formal competencies or guidelines, the nurses expressed how they relied on their personal empathy and understanding of the relatives' situation combined with personal competencies and experiences, leaving the care provided to the BID persons and their relatives to be rather subjective and random.

So it actually comes to my mind… how little grief and crisis are talked about in an ED … Where I have been used to… hospital departments have a routine … there is a lot of talk… we always try to open the window [symbolic of letting the spirit/soul away], and whatever it may be… I don't actually hear that it is something that is spoken about here (Mette, 47 years).

The nurses described how planning care based on only personal experiences unambiguously contrasted with the usual organization of ED care, which was highly standardized, continuously trained, and based on various decision‐making tool systems, action cards and algorithms. However, caring for BID persons and their relatives required other education and competencies than those, in the ED, highly valued ‘life‐saving skills’.

In addition, the nurses described how they went back and forth between BID situations with dramatic emotions and other tasks like caring for a patient with a sprained ankle. In the world of ED nursing, a sprained ankle is a frequent, minor and harmless injury, whereas caring for BID persons is highly complex and requires other competencies and skills. Caring for BID persons who, for example, are accompanied by relatives in acute grief, have been dead for hours or days, are missing body parts, or are severely injured in other ways, affects the nurses' perception of normality and care routines. Personal skills, own life circumstances and former experiences dealing either professionally or personally with death were highlighted as important to be able to handle and pending between different care situations.

### 4.5 Navigating professionalism and empathy

The nurses described how they perceived BID care as being able to relate to grief and loss professionally and having the time to be with and support the BID relatives without interruption from other tasks. ‘To stay in the task—and own it’ (Mia, 37 years) as one formulated it. Nonetheless, the nurses also acknowledged the emotional difficulty of such dedication, noting that there were moments when the emotional demands of the role stretched them to the edges of their personal and professional capacities.

Because you are tired. Completely drained of energy. And also, because even though we are professionals, you can familiarize yourself with that there are two parents and a brother who have lost someone dear. And losing a child, you just shouldn't. It is meaningless. So, we are also personally involved in some way (Julie, 41 years).

In those cases, it was described that trust in colleagues was important so that it was possible to say, ‘I'm not ok’. Some described that they and their colleagues were really good at supporting and debriefing each other, while others had the opposite experience.

The nurses have different and competing perspectives on how to navigate and balance between personal and professional conduct concerning, for example, crying with relatives. Some said they cried with the relatives; others considered this unprofessional. Some nurses preferred not to care for BID persons who were in a similar life situation as themselves because it made them feel vulnerable and increased the risk that they could not ‘control’ or hide their emotional reactions.

… had many deceased children … my child was the same age, so my [emotional]backpack was full … I just started crying… our job is to be calm, professional and guide relatives through the shock and grief that it is. It is not our job to show feelings, but we must be, what can you say, able to calmly guide the process and support the relatives (Maria, 56 years).

Other nurses were indifferent and did not mind.

… It's okay to cry with the relatives. It's okay to show that we are affected too. Because that's what you become. … Some are very affected when it is children … I am equally affected when it is the relative of the 75‐year‐old woman who loses her husband, and they are the only two who have done everything for each other. And now she has no one else. It is so infinitely sad. (Mona, 60 years).

The nurses described how emotional resilience was a required skill, being able to stay with the BID relatives and contain and control a variety of emotions during their visit to the ED. Directly asked, the nurses stated that they wished they had the opportunity to follow up with the relatives, for example, by calling them after a week or two. However, this was most often opted out by the nurses due to busyness and a lack of resources. Consequently, the nurses were deprived of any kind of feedback on their delivered care.

## 5 DISCUSSION

We explored how nurses experienced caring for BID persons and their relatives in EDs and identified several obstacles within ED settings that hindered the care for BID persons and their relatives. This study illustrated how the nurses were navigating the complexities of providing BID care in constrained ED environments. However, it also reveals that the structural framework and the conditions under which ED nurses operated to fulfil this task were often unreasonable.

Our findings illustrate a dichotomy between the high‐paced, outcome‐focused nature of emergency care and the low‐paced, holistic, person‐centred approach essential for palliative care and bereavement support (De Campos et al., 2022). Although the nurses acknowledged the significance of attending to BID persons and their relatives, this aspect of care frequently remained unrecognized. This lack of recognition not only constrains the development and valorization of specialized care for BID persons and their relatives but may also signify a deeper professional reluctance towards engaging with death and bereavement within the lifesaving context of EDs. Although ED nurses want to provide high quality care for dying patients and their relatives, several studies point to a deficiency in palliative care education and training and environmental barriers in the EDs (Giles et al., 2019; Green et al., 2017; Sausman et al., 2023b).

In our study, the ED nurses relied on personal experiences due to the absence of education or guidelines regarding BID care. While empathy and experiential learning are invaluable, the lack of structured training and support for nurses in dealing with BID situations can lead to variability in care practices, threaten care quality and potentially affect the grieving process of the relatives (Wilson et al., 2022). This finding calls for the integration of training in care for BID persons and their relatives within ED nursing education and continuous professional development programs. However, providing care for BID persons and their relatives requires some additional skills, competencies and knowledge than providing palliative care. Although ED nurses can draw on their education and experience within palliative care, the circumstances and context surrounding the care of BID persons are unique and differ significantly from other care settings (Aquino et al., 2022b; Giles et al., 2019; Prachanukool et al., 2023).

Our findings illuminate the necessity for an evidence‐based holistic approach that encompasses not only the short‐term technical and standardized aspects of care for BID persons and their relatives but also the long‐term emotional, spiritual and social dimensions that align with palliative care philosophy. Adapting such a philosophy within the ED could mitigate some of the challenges identified, facilitating a more compassionate and comprehensive care model for BID persons and their relatives. As this study and our previous study illustrate (Bove et al., 2022), care for BID persons and their relatives depends on knowledge about both ED care, palliative care and trauma care.

To provide care for BID persons and their relatives, nurses need to be able to merge and apply this knowledge to the unique ED context of BID persons and their relatives. However, evidence‐based knowledge of how to do this is demanded and an area highly recommended for further research.

Traditionally, the responsibility for providing care to BID persons and their relatives has been assigned to EDs, a practice likely rooted in longstanding traditions and customs. This practice has remained unaltered, probably because the responsibility has been invisible and never acknowledged as significant by either nurses or other healthcare professionals, such as physicians and leaders. With EDs encountering escalating activity, complexity and a need for more specialized skills in acute, instrumental nursing tasks, the provision of care for the BID population increasingly misaligns with the acute care context. This incongruence suggests a need to reassess the responsibility of BID care within EDs.

The largely invisible nature of BID care exacerbates the issue, leading to a void in quality assurance and a stark absence of guidelines for delivering effective care in these circumstances. Furthermore, the absence of feedback mechanisms, since BID persons and their grieving relatives are unable to provide direct feedback, and the potential complications of pathological grief leave nurses without any direct assessment and feedback of the care provided.

Our findings resonate with existing literature on moral distress among nurses, where the inability to provide adequate care due to external constraints leads to professional and emotional discomfort (Rushton, 2016). The unpredictable nature of BID cases, coupled with the existing pressures of emergency care, exacerbates this distress, underscoring the need for organizational and structural support for nurses in these situations. Furthermore, burnout and stress are frequent among nurses, especially among ED nurses (Alsalmi & Alilyyani, 2023). High workload and lack of time to perform tasks, fear of making mistakes, high job and psychological demands, and poor supervisor/leader support are described as some of the risk factors for burnout and stress among ED nurses (Dall'Ora et al., 2020).

We do not question that it should be the responsibility of nurses to care for the BID relatives, as it in every way draws on core nursing competencies. On the contrary, we perceive caring for BID relatives as highly complex nursing. Care for BID persons and their relatives is not something that just has to be solved as one of many tasks in a busy ED and by ED nurses without the necessary time, competencies, or dedication. Altogether, these scenarios underscore the urgent need for establishing clear guidelines and training to address the unique needs of the BID population and their relatives, ensuring that nurses are educated and given the time and support needed to deliver compassionate and competent care. An alternative solution is to remove the responsibility for BID care from the EDs.

### 5.1 Limitations and strengths

The data collection strategy deviated from the protocol and our original intent of conducting focus groups (Bove et al., 2020). Due to the busy and unpredictable nature of tasks in an ED, it was not practically feasible to assemble a sufficient number of nurses for focus group interviews. Consequently, we opted to conduct individual interviews instead.

One notable limitation of our study is the potential sampling bias resulting from our use of convenience sampling. This method may have led to the inclusion of ED nurses with specific perceptions or attitudes towards the care of BID persons and their relatives. It is also a weakness that there has been no patient and public involvement, either in the design of the study or in the development of the interview topic guide.

Despite the limitations of convenience sampling, a significant strength of our study is the recruitment of participants from seven different EDs. This multi‐site approach contributes to a diverse range of experiences, attitudes and practices among the included participants, enhancing the potential transferability of our findings to various healthcare settings.

### 5.2 Recommendations for further research

Given the relatively invisible and underrecognized nature of the care for BID persons and their relatives within EDs, there exists a significant knowledge gap both nationally and globally. This gap underscores the urgent need for further research. Key areas of inquiry include the development and structuring of educational programmes specifically tailored for ED nurses who provide care for BID persons and their relatives. Such programmes should focus on both the organizational aspects and the substantive content necessary to enhance this area of care and the significance of the physical environment. Additionally, exploring alternative models for the delivery of care to BID persons and their relatives represents a critical field of study. Investigating whether palliative care departments or teams, specially trained ED teams, or entirely novel approaches could more effectively manage the care of BID persons and their relatives is essential for advancing and improving care for BID persons and their families.

### 5.3 Implications for policy and practice

The findings from this study have significant implications for clinical practice and health policy. First, there is a clear need for policy frameworks that recognize and support the role of ED nurses in providing care for BID persons and their relatives. Second, enhancing the physical environment of EDs to accommodate the support needs of grieving relatives could substantially improve the quality of care for this population. Finally, developing and implementing formal guidelines and training programmes for ED nurses caring for BID person and their relatives could ensure a more standardized and empathetic approach to BID situations.

## 6 CONCLUSION

EDs, inherently designed for acute interventions, posed unique challenges in providing care to BID persons and their grieving relatives. This study highlights the contrast between the high‐paced, outcome‐focused nature of emergency care and the nuanced, empathetic approach required for caring for BID persons and their relatives. The unrecognized nature of this task indicates a systemic undervaluation of caring for BID persons and their relatives within the ED context, potentially increasing the risk of burnout and moral stress among ED nurses. This not only impedes the acknowledgment of this vital aspect of care but also hinders the development and recognition of the nursing competencies essential for providing comprehensive care for BID persons and their relatives.

## AUTHOR CONTRIBUTIONS

D.G.B., S.F.H. and H.T. made substantial contributions to the conception and design of the study. S.S. conducted the interviews. All authors engaged in the analysis and interpretation of the data. D.G.B. drafted the initial manuscript. S.S., D.G.B., S.F.H. and H.T. were actively involved in refining the manuscript and revising it critically for important intellectual content. All authors agree to be accountable for all aspects of the work, ensuring that questions related to the accuracy or integrity of any part of the work are appropriately investigated and resolved. All authors have agreed on the final version and meet the ICMJE criteria for authorship.

## FUNDING INFORMATION

This work was supported by a grant given to the first author from The Lundbeck Foundation.

## CONFLICT OF INTEREST STATEMENT

The authors have no conflicts of interest to declare.

## ETHICS STATEMENT

The study was conducted in accordance with the Declaration of Helsinki. According to Danish law and The Ethics Committee (https://en.nationaltcenterforetik.dk/) in Denmark, no formal approval applies to qualitative studies in general.

## PARTICIPANTS CONSENT STATEMENT

All participants (nurses) were informed both in writing and verbally about the aim of the study and its voluntary nature. All participants signed an informed consent from before the interview.

## Data Availability

The data are not publicly available, but data in the form of transcriptions can be made available on a reasonable request to the corresponding author. All data material is in Danish.

## References

[jan16371-bib-0001] Alarhayem, A. Q. , Cohn, S. M. , Muir, M. T. , Myers, J. G. , Fuqua, J. , & Eastridge, B. J. (2017). Organ donation, an unexpected benefit of aggressive resuscitation of trauma patients presenting dead on arrival. Journal of the American College of Surgeons, 224(5), 926–932. 10.1016/j.jamcollsurg.2017.02.012 28263857

[jan16371-bib-0056] Alsalmi, M. , & Alilyyani, B. (2023). The role of authentic leadership in nurses’ stress and burnout in emergency departments. Leadership in Health Services, 37(1), 147–158. 10.1108/lhs-01-2023-0005 37606378

[jan16371-bib-0002] Aquino, J. , Crilly, J. , & Ranse, K. (2022). The end‐of‐life care practices of emergency care nurses and the factors that influence these practices: An integrative review. International Emergency Nursing, 63, 101168. 10.1016/j.ienj.2022.101168 35584560

[jan16371-bib-0003] Bayuo, J. , Agbeko, A. E. , Acheampong, E. K. , Abu‐Odah, H. , & Davids, J. (2022). Palliative care interventions for adults in the emergency department: A review of components, delivery models, and outcomes. Academic Emergency Medicine, 29(11), 1357–1378. 10.1111/acem.14508 35435306

[jan16371-bib-0004] Beckstrand, R. L. , Corbett, E. M. , Macintosh, J. L. B. , Luthy, K. E. B. , & Rasmussen, R. J. (2019). Emergency Nurses' Department design recommendations for improved end‐of‐life care. Journal of Emergency Nursing, 45(3), 286–294. 10.1016/j.jen.2018.05.014 30017423

[jan16371-bib-0005] Beckstrand, R. L. , Rasmussen, R. J. , Luthy, K. E. , & Heaston, S. (2012). Emergency Nurses' perception of department design as an obstacle to providing end‐of‐life care. Journal of Emergency Nursing, 38(5), e27–e32. 10.1016/j.jen.2011.12.019 22595683

[jan16371-bib-0006] Bove, D. G. , Herling, S. F. , Sørensen, N. , Gjersøe, P. , & Timm, H. (2022). A qualitative study of the experiences of relatives to brought in dead persons in an emergency department. Journal of Advanced Nursing, 78, 4165–4176. 10.1111/jan.15384 35976705 PMC9804706

[jan16371-bib-0007] Bove, D. G. , Rosted, E. , Prip, A. , Jellington, M. O. , Timm, H. , & Herling, S. F. (2020). How to care for the brought in dead and their relatives. A qualitative study protocol based on interpretive description. Journal of Advanced Nursing, 76(7), 1794–1802. 10.1111/jan.14353 32180240

[jan16371-bib-0008] Bove, D. G. , Sørensen, N. , Timm, H. , Herling, S. F. , & Gjersøe, P. (2021). Patient characteristics of persons dead on arrival received in a Danish emergency department: A retrospective review of health records. Journal of Emergency Nursing, 47(4), 582–589. e1. 10.1016/j.jen.2021.01.007 33642054

[jan16371-bib-0009] Bradley, C. (2021). Family presence and support during resuscitation. Critical Care Nursing Clinics of North America, 33(3), 333–342. 10.1016/j.cnc.2021.05.008 34340794

[jan16371-bib-0010] Braun, V. , & Clarke, V. (2006). Using thematic analysis in psychology. Qualitative Research in Psychology, 3(2), 77–101. 10.1191/1478088706qp063oa

[jan16371-bib-0011] Braun, V. , & Clarke, V. (2019). Reflecting on reflexive thematic analysis. In Qualitative research in sport, exercise and health (Vol. 11, pp. 589–597). Routledge. 10.1080/2159676X.2019.1628806

[jan16371-bib-0012] Byrne, D. (2022). A worked example of Braun and Clarke's approach to reflexive thematic analysis. Quality and Quantity, 56(3), 1391–1412. 10.1007/s11135-021-01182-y

[jan16371-bib-0013] Carlsson, N. , Alvariza, A. , Axelsson, L. , Bremer, A. , & Årestedt, K. (2022). Grief reactions in relation to professional and social support among family members of persons who died from sudden cardiac arrest: A longitudinal survey study. Resuscitation Plus, 12, 100318. 10.1016/j.resplu.2022.100318 36299826 PMC9589205

[jan16371-bib-0014] Cavaye, J. , & Watts, J. H. (2014). An integrated literature review of death education in pre‐registration nursing curricula: Key themes. International Journal of Palliative Care, 2014, 1–19. 10.1155/2014/564619

[jan16371-bib-0015] Chang, A. , Espinosa, J. , Lucerna, A. , & Parikh, N. (2022). Palliative and end‐of‐life care in the emergency department. Clinical and Experimental Emergency Medicine, 9(3), 253–256. 10.15441/ceem.22.341 36221963 PMC9561206

[jan16371-bib-0054] Clarke, V. , & Braun, V. (2017). Thematic analysis. The Journal of Positive Psychology, 12(3), 297–298. 10.1080/17439760.2016.1262613

[jan16371-bib-0057] Dall’Ora, C. , Ball, J. , Reinius, M. , & Griffiths, P. (2020). Burnout in nursing: a theoretical review. Human Resources for Health, 18(1). 10.1186/s12960-020-00469-9 PMC7273381 32503559

[jan16371-bib-0016] De Campos, A. P. , Levoy, K. , Pandey, S. , Wisniewski, R. , Dimauro, P. , Ferrell, B. R. , & Rosa, W. E. (2022). Integrating palliative care into nursing care. American Journal of Nursing, 122(11), 40–45. 10.1097/01.NAJ.0000897124.77291.7d PMC9889110 36261904

[jan16371-bib-0017] Díaz‐Cortés, M. D. M. , Granero‐Molina, J. , Hernández‐Padilla, J. M. , Pérez Rodríguez, R. , Correa Casado, M. , & Fernández‐Sola, C. (2018). Promoting dignified end‐of‐life care in the emergency department: A qualitative study. International Emergency Nursing, 37, 23–28. 10.1016/j.ienj.2017.05.004 28655591

[jan16371-bib-0018] Durojaiye, A. , Ryan, R. , & Doody, O. (2023). Student nurse education and preparation for palliative care: A scoping review. PLoS One, 18(7), e0286678. 10.1371/journal.pone.0286678 37399170 PMC10317240

[jan16371-bib-0019] Eriksson, J. , Gellerstedt, L. , Hillerås, P. , & Craftman, Å. G. G. (2018). Registered nurses' perceptions of safe care in overcrowded emergency departments. Journal of Clinical Nursing, 27(5–6), e1061–e1067. 10.1111/jocn.14143 29076280

[jan16371-bib-0020] Fernández‐Sola, C. , Cortés, M. M. D. , Hernández‐Padilla, J. M. , Torres, C. J. A. , Terrón, J. M. M. , & Granero‐Molina, J. (2017). Defining dignity in end‐of‐life care in the emergency department. Nursing Ethics, 24(1), 20–32. 10.1177/0969733015604685 26391693

[jan16371-bib-0021] Gage, C. H. , Stander, C. , Gwyther, L. , & Stassen, W. (2023). Emergency medical services and palliative care: A scoping review. BMJ Open, 13(3), e071116. 10.1136/bmjopen-2022-071116 PMC10030966 36927584

[jan16371-bib-0022] Giles, T. M. , Hammad, K. , Breaden, K. , Drummond, C. , Bradley, S. L. , Gerace, A. , & Muir‐Cochrane, E. (2019). Nurses' perceptions and experiences of caring for patients who die in the emergency department setting. International Emergency Nursing, 47, 100789. 10.1016/j.ienj.2019.100789 31495727

[jan16371-bib-0023] Green, E. , Ward, S. , Brierley, W. , Riley, B. , Sattar, H. , & Harris, T. (2017). “They Shouldn't Be coming to the ED, should they?”: A descriptive service evaluation of why patients with palliative care needs present to the emergency department. American Journal of Hospice & Palliative Medicine, 34(10), 984–990. 10.1177/1049909116676774 27903774

[jan16371-bib-0024] Hajradinovic, Y. , Tishelman, C. , Lindqvist, O. , & Goliath, I. (2018). Family members experiences of the end‐of‐life care environments in acute care settings—a photo‐elicitation study. International Journal of Qualitative Studies on Health and Well‐Being, 13(1), 1511767. 10.1080/17482631.2018.1511767 30176152 PMC6127834

[jan16371-bib-0025] Heeke, C. , Kampisiou, C. , Niemeyer, H. , & Knaevelsrud, C. (2017). A systematic review and meta‐analysis of correlates of prolonged grief disorder in adults exposed to violent loss. European Journal of Psychotraumatology, 8(sup6). 1‐20 10.1080/20008198.2019.1583524 PMC6442112 30949303

[jan16371-bib-0026] Hogan, K. A. , Fothergill‐Bourbonnais, F. , Brajtman, S. , Phillips, S. , & Wilson, K. G. (2016). When someone dies in the emergency department: Perspectives of emergency nurses. Journal of Emergency Nursing, 42(3), 207–212. 10.1016/j.jen.2015.09.003 26435352

[jan16371-bib-0027] Howarth, G. (2010). Viewing the body after a traumatic death. BMJ, 340(apr30 2), c2301. 10.1136/bmj.c2301 20435645

[jan16371-bib-0028] Hunter, D. , McCallum, J. , & Howes, D. (2018a). Compassion in emergency departments. Part 2: Barriers to the provision of compassionate care. Emergency Nurse, 26(3), 28–33. 10.7748/en.2018.e1775 30047712

[jan16371-bib-0029] Hunter, D. , McCallum, J. , & Howes, D. (2018b). Compassion in emergency departments. Part 3: Enabling and supporting delivery of compassionate care. Emergency Nurse, 26(4), 28–31. 10.7748/en.2018.e1776 30299006

[jan16371-bib-0052] Martí‐García, C. , Fernández‐Férez, A. , Fernández‐Sola, C. , Pérez‐Rodríguez, R. , Esteban‐Burgos, A. A. , Hernández‐Padilla,, J. M. , & Granero‐Molina, J. (2023). Patients‘ experiences and perceptions of dignity in end‐of‐life care in emergency departments: A qualitative study. Journal of Advanced Nursing, 79(1), 269–280. 10.1111/jan.15432 36062865 PMC10087743

[jan16371-bib-0030] Mayer, D. D. (2017). Improving the support of the suddenly bereaved. Current Opinion in Supportive and Palliative Care, 11(1), 1–6. 10.1097/SPC.0000000000000253 28009649

[jan16371-bib-0031] Merlevede, E. , Spooren, D. , Henderick, H. , Portzky, G. , Buylaert, W. , Jannes, C. , Calle, P. , Van Staey, M. , De Rock, C. , Smeesters, L. , Michem, N. , & Van Heeringen, K. (2004). Perceptions, needs and mourning reactions of bereaved relatives confronted with a sudden unexpected death. Resuscitation, 61(3), 341–348. 10.1016/j.resuscitation.2004.01.024 15172714

[jan16371-bib-0032] Mughal, S. , Azhar, Y. , Mahon, M. M. , & Siddiqui, W. J. (2024). Grief reaction and prolonged grief disorder. 29939609

[jan16371-bib-0033] Murnane, S. , Purcell, G. , & Reidy, M. (2023). Death, dying and caring: Exploring the student nurse experience of palliative and end‐of‐life education. British Journal of Nursing, 32(11), 526–531. 10.12968/bjon.2023.32.11.526 37289708

[jan16371-bib-0034] Nordt, S. P. , Ryan, J. M. , Kelly, D. , Kutubi, A. , Saleh, R. , Quinn, C. , Al Kharusi, T. , & Tiernan, E. J. (2023). Palliative care patient emergency department visits at tertiary university‐based emergency department in Ireland. The American Journal of Emergency Medicine, 66, 76–80. 10.1016/j.ajem.2023.01.035 36736062

[jan16371-bib-0035] Omerov, P. , Steineck, G. , Nyberg, T. , Runeson, B. , & Nyberg, U. (2014). Viewing the body after bereavement due to suicide: A population‐based survey in Sweden. PLoS One, 9(7), e101799. 10.1371/journal.pone.0101799 24999660 PMC4085007

[jan16371-bib-0036] Pitman, A. L. , Stevenson, F. , Osborn, D. P. J. , & King, M. B. (2018). The stigma associated with bereavement by suicide and other sudden deaths: A qualitative interview study. Social Science & Medicine, 198, 121–129. 10.1016/j.socscimed.2017.12.035 29316512 PMC5884304

[jan16371-bib-0037] Powers, K. A. (2017). Educational interventions to improve support for family presence during resuscitation. Dimensions of Critical Care Nursing, 36(2), 125–138. 10.1097/DCC.0000000000000228 28151791

[jan16371-bib-0038] Prachanukool, T. , George, N. , Bowman, J. , Ito, K. , & Ouchi, K. (2023). Best practices in end of life and palliative care in the Emergency Department. Clinics in Geriatric Medicine, 39(4), 575–597. 10.1016/j.cger.2023.05.011 37798066 PMC11300921

[jan16371-bib-0055] Rushton, C. H. , Caldwell, M. & Kurtz, M. (2016). CE. AJN, American Journal of Nursing, 116(7), 40–49. 10.1097/01.naj.0000484933.40476.5b 27294668

[jan16371-bib-0039] Sausman, J. , Arif, A. , Young, A. , MacArtney, J. , Bailey, C. , Rajani, J. , & Burt, R. (2023). Healthcare professionals' perspectives of the management of people with palliative care needs in the emergency department of a UK hospital. BMC Palliative Care, 22(1), 129. 10.1186/s12904-023-01248-8 37670312 PMC10481573

[jan16371-bib-0040] Sefton, C. , Keen, S. , Tybout, C. , Lin, F.‐C. , Jiang, H. , Joodi, G. , Williams, J. G. , & Simpson, R. J. (2023). Characteristics of sudden death by clinical criteria. Medicine, 102(16), e33029. 10.1097/MD.0000000000033029 37083784 PMC10118332

[jan16371-bib-0041] Şimşek Arslan, B. , & Buldukoğlu, K. (2023). Grief rituals and grief reactions of bereaved individuals during the COVID‐19 pandemic. OMEGA—Journal of Death and Dying, 87(4), 1293–1307. 10.1177/00302228211037591 34344252

[jan16371-bib-0042] Szuhany, K. L. , Malgaroli, M. , Miron, C. D. , & Simon, N. M. (2021). Prolonged grief disorder: Course, diagnosis, assessment, and treatment. Focus, 19(2), 161–172. 10.1176/appi.focus.20200052 34690579 PMC8475918

[jan16371-bib-0043] Taheri‐Ezbarami, Z. , Jafaraghaee, F. , Sighlani, A. K. , & Mousavi, S. K. (2024). Core components of end‐of‐life care in nursing education programs: A scoping review. BMC Palliative Care, 23(1), 82. 10.1186/s12904-024-01398-3 38549106 PMC10976691

[jan16371-bib-0044] Tarassenko, S. (2010). Benefits of viewing the body. BMJ, 340(may25 3), c2757. 10.1136/bmj.c2757 20501563

[jan16371-bib-0045] Thompson Burdine, J. , Thorne, S. , & Sandhu, G. (2021). Interpretive description: A flexible qualitative methodology for medical education research. Medical Education, 55(3), 336–343. 10.1111/medu.14380 32967042

[jan16371-bib-0046] Thorne, S. (2016). Interpretive description: Qualitative research for applied practice (2nd ed.). Routledge.

[jan16371-bib-0047] Tobin, G. A. , & Begley, C. M. (2004). Methodological rigour within a qualitative framework. Journal of Advanced Nursing, 48(4), 388–396. 10.1111/j.1365-2648.2004.03207.x 15500533

[jan16371-bib-0048] Tong, A. , Sainsbury, P. , & Craig, J. (2007). Consolidated criteria for reporting qualitative research (COREQ): A 32‐item checklist for interviews and focus groups. International Journal for Quality in Health Care, 19(6), 349–357. 10.1093/intqhc/mzm042 17872937

[jan16371-bib-0049] Wilson, D. M. , Darko, E. M. , Kusi‐Appiah, E. , Roh, S. J. , Ramic, A. , & Errasti‐Ibarrondo, B. (2022). What exactly is “complicated” grief? A scoping research literature review to understand its risk factors and prevalence. OMEGA—Journal of Death and Dying, 86(2), 471–487. 10.1177/0030222820977305 33259275

[jan16371-bib-0050] World Health Organisation (WHO) . (2024). Palliative care. Https://Www.Who.Int/Health‐Topics/Palliative‐Care. https://www.who.int/health‐topics/palliative‐care

[jan16371-bib-0051] Zavrou, R. , Charalambous, A. , Papastavrou, E. , Koutrouba, A. , & Karanikola, M. (2023). Trying to keep alive a non‐traumatizing memory of the deceased: A meta‐synthesis on the interpretation of loss in suicide‐bereaved family members, their coping strategies and the effects on them. Journal of Psychiatric and Mental Health Nursing, 30(2), 182–207. 10.1111/jpm.12866 35996970

